# Association between elevated serum alanine aminotransferase and cardiometabolic risk factors in rural Chinese population: a cross-sectional study

**DOI:** 10.1186/s12872-015-0060-y

**Published:** 2015-07-10

**Authors:** Shuang Chen, Xiaofan Guo, Xingang Zhang, Shasha Yu, Hongmei Yang, Mohan Jiang, Guozhe Sun, Yingxian Sun

**Affiliations:** Department of Cardiology, The First Affiliated Hospital of China Medical University, 155 Nanjing North Street, Heping District, 110001 Shenyang, People’s Republic of China

**Keywords:** Alanine aminotransferase, Cardiometabolic risk factors, Rural Chinese Population, Sex-related differences

## Abstract

**Background:**

Elevated alanine aminotransferase (ALT) levels may be associated with metabolic syndrome and cardiovascular diseases. This study aimed to investigate the association between elevated ALT levels and cardiometabolic risk factors in a rural Chinese population.

**Methods:**

This was a cross-sectional study conducted from July 2012 to August 2013, including 11,573 subjects (5,357 men and 6,216 women) aged ≥35 years in rural areas of Liaoning Province. A physical examination was performed and metabolic indicators were examined under standard protocols. Subjects were divided into those with elevated ALT levels (>40U/L) and those with normal ALT levels (≤40U/L).

**Results:**

Participants with elevated ALT levels had higher levels of almost all cardiometabolic risk factors than those with normal ALT levels. In individuals with elevated ALT levels, weight, height, waist circumference, and body mass index (BMI), which are indicators for general and abdominal obesity, were significantly higher (p < 0.001) than those in individuals with normal ALT levels. There was no significant difference in race, current smoking, or physical activity between the two groups. Other cardiometabolic risk factors, such as systolic blood pressure, diastolic blood pressure, and fasting plasma glucose, TC, TG, low-density lipoprotein cholesterol, and serum uric acid levels, were higher in participants with elevated ALT levels than in those with normal ALT levels. Logistic regression analysis showed that male sex, younger age, and the presence of high TC, high TG, and low high-density lipoprotein cholesterol levels, current smoking status, BMI ≥25 kg/m^2^, abdominal obesity, hyperuricemia, and HtgW phenotype were significantly (p < 0.05) associated with elevated ALT levels. Sex-related differences were also investigated. For men, hypertension (OR 1.33, 95 % CI 1.08–1.62), high TC levels (OR 1.63, 95 % CI 1.23–2.17), high TG levels (OR 1.62, 95 % CI 1.25–2.09), BMI ≥25 kg/m^2^ (OR 1.52, 95 % CI 1.07–2.18), and hyperuricemia (OR 1.92, 95 % CI 1.52–2.40) were significantly (p < 0.05) related to elevated serum ALT levels, but this was not observed in women.

**Conclusions:**

There are significant relationships of elevated ALT levels with cardiometabolic risk factors and several sex-related differences in rural Chinese. Elevated serum ALT levels are associated with a worse cardiac risk profile.

## Background

Alanine aminotransferase (ALT) is considered as a sensitive indicator of liver cell injury [1, 2]. Because ALT is closely related to fat accumulation in the liver [3], it is also commonly considered as a surrogate marker for nonalcoholic fatty liver disease (NAFLD) in some epidemiological studies [4, 5]. Recent findings have shown that nonalcoholic fatty liver disease is strongly associated with obesity, metabolic syndrome, and diabetes mellitus, as well as cardiovascular events [6–9]. Several studies have indicated that elevated serum ALT levels are associated with age, sex, obesity, waist circumference, fasting blood glucose levels, and serum triglyceride levels [10, 11]. Therefore, patients with cardiometabolic risk factors usually have elevated serum ALT activity, and serum ALT levels are commonly used to monitor liver function in people with metabolic disorders. However, there are limited data linking elevated serum ALT levels and physical activity [12]. The HtgW phenotype is represented by the simultaneous presence of elevated serum triglyceride levels and an increased waist circumference [13], and this phenotyope is a major determinant of cardiometabolic risk among Turks [14].

Several large-scale, population-based surveys have evaluated serum ALT levels in developed countries, but there are limited data from developing countries. In China, previous studies on ALT and cardiometabolic risk factors had limitations in the study design and a small sample size. Large-scale, population-based, cross-sectional surveys addressing the associations between elevated serum alanine aminotransferase levels and cardiometabolic risk factors in a rural Chinese population have not been performed. Therefore, we conducted this study to investigate 1) the association between elevated ALT levels and cardiometabolic risk factors in a rural Chinese population and 2) differences in cardiometabolic characteristics associated with elevated ALT levels in both sexes.

## Methods

### Study population

Liaoning Province is located in Northeast China. From July 2012 to August 2013, a representative sample aged ≥35 years was selected to describe the prevalence, incidence and natural history of cardiovascular risk factors in rural areas of Liaoning Province, which is called Northeast China Rural Cardiovascular Health Study (NCRCHS). The study adopted a multi-stage, stratified randomly cluster-sampling scheme. In the first stage, 3 counties (Dawa, Zhangwu, and Liaoyang County) were selected from the eastern, southern, and northern region of Liaoning province. In the second stage, one town was randomly selected from each county (a total of 3 towns). In the third stage, 8–10 rural villages from each town were randomly selected (a total of 26 rural villages). All the eligible permanent residents aged ≥ 35 years from each village were invited to attend the study (a total of 14,016 participants). Of those, 11,956 participants agreed and completed the present study and the response rate was 85.3 %. The study was approved by the Ethics Committee of China Medical University (Shenyang, China). All procedures were performed in accordance with the ethical standards. Written consent was obtained in all participants after they had been informed of the objectives, benefits, medical items and confidentiality agreement of personal information. If the participants were illiterate, we obtained the written informed consents from their proxies. In this report, we used data of baseline and only participants with a complete set of data regarding the variables analyzed in the study were included, making a final sample size of 11,573 (5,357 men and 6,216 women).

### Data collection and measurements

Data was collected during a single clinic visit by cardiologists and trained nurses using a standard questionnaire by face-to-face interview. Before the survey was performed, we invited all eligible investigators to attend the organized training. The training contents included the purpose of this study, how to administer the questionnaire, the standard method of measurement, the importance of standardization, and the study procedures. A strict test was evaluated after this training, only those who scored perfectly on the test could become investigators. During data collection, our inspectors had further instructions and support.

Data on demographic characteristics, lifestyle risk factors, family income, medical history, were obtained by interview with a standardized questionnaire. There was a central steering committee with a subcommittee for quality control. Educational level was divided into primary school or below, middle school and high school or above. Family income was classified as ≤5000, 5000–20000 and >20000 CNY/year.

According to American Heart Association protocol, blood pressure was measured three times at 2-min intervals after at least 5 min of rest using a standardized automatic electronic sphygmomanometer (HEM-741C; Omron, Tokyo, Japan). The calibration of the Omron device was checked using a standard mercury sphygmomanometer every month by two doctors according to the British Hypertension Society protocol [15]. The participants were advised to avoid caffeinated beverages and exercise for at least 30 min before the measurement. During the measurement, the participants were seated with the arm supported at the level of the heart. The mean of three BP measures was calculated and used in all analyses.

Weight and height were measured to the nearest 0.5 kg and 0.1 cm respectively with the participants in light weight clothing and without shoes. WC was measured at the midpoint between the lower rib and upper margin of the iliac crest using a non-elastic tape (to the nearest 0.1 cm), with the participants standing at the end of normal expiration. The body mass index (BMI) was calculated as weight in kilograms divided by the square of the height in meters.

Fasting blood samples were collected in the morning after at least 12 h of fasting for all participants. Blood samples were obtained from an Antecubital vein into Vacutainer tubes containing EDTA. Serum was subsequently isolated from the whole blood, and all serum samples were frozen at −20 °C for testing at a central, certified laboratory. Fasting plasma glucose (FPG), total cholesterol (TC), low-density lipoprotein cholesterol (LDL-C), high-density lipoprotein cholesterol (HDL-C), triglyceride (TG), Serum uric acid(SUA) and other routine blood biochemical indexes were analyzed enzymatically on an Olympus AU640 autoanalyzer (Olym-pus, Kobe, Japan). All laboratory equipment was calibrated and blinded duplicate samples were used.

### Definitions

According to JNC-7 report [16], hypertension was defined as SBP ≥140 mm Hg and/or DBP ≥90 mm Hg and/or use of antihypertensive medications. Diabetes mellitus was diagnosed according to the WHO criteria [17]: FPG ≥7 mmol/L (126 mg/dL) and/or being on treatment for diabetes. High TC was defined as TC ≥6.21 mmol/L (240 mg/dL). High TG was defined as TG ≥2.26 mmol/L (200 mg/dL). High LDL-C was de fined as LDL-C ≥4.16 mmol/L (160 mg/dL). Low HDL-C was defined as HDL-C <1.03 mmol/L (40 mg/dL) [18]. Hyperuricemia was defined as serum uric acid ≥416 μmol/l in men and ≥357 μmol/l in women according to guidelines [19]. BMI was categorized into 3 groups as normal (BMI <25 kg/m^2^), overweight (25 ≤ BMI <30 kg/m^2^) and obesity (BMI ≥30 kg/m^2^), according to the World Health Organization (WHO) criteria [20]. Abdominal obesity was defined as WC ≥88 cm for females and WC ≥102 cm for males [21]. HtgW phenotype was defined as fasting triglycerides ≥1.7 mM combined with abdominal obesity. Elevated serum ALT level was defined as ALT >40 U/L [22].

The smoking and alcohol consumption status were also surveyed. Self-reported smoking and drinking were obtained from the questionnaire. Current smokers or drinkers were defined as people who were currently smoking or drinking. Former and never smokers or drinkers were treated as the other category.

Physical activity included occupational and leisure-time physical activity. A detailed description of the methods for assessing physical activity has been presented elsewhere. Occupational and leisure-time physical activity were merged and regrouped into the following three categories: 1) low—subjects who reported light levels of both occupational and leisure-time physical activity, 2) moderate—subjects who reported moderate or high levels of either occupational or leisure-time physical activity and 3) high—subjects who reported a moderate or high level of both occupational and leisure-time physical activity [23].

### Statistical analysis

Descriptive statistics were calculated for all the variables, including continuous variables which were normally distributed (reported as mean values and standard deviations) and categorical variables (reported as numbers and percentages). Differences among ALT and sex categories were evaluated using Student’s *t*-test, or the *χ*2-test as appropriate. Multivariate logistic regression analyses were used to identify the association between elevated ALT and caridometabolic risk factors with odds ratios (ORs) and corresponding 95 % confidence intervals (CIs) calculated. All the statistical analyses were performed using SPSS version 17.0 software (SPSS Inc, Chicago, Illinois, USA), and *P* values less than 0.05 were considered to be statistically significant.

## Results

### Baseline characteristics of the study population

Table 1 shows baseline data according to serum ALT levels and sex. A total of 11,573 subjects (5,357 men and 6,216 women) participated in the study. The general characteristics of the study population with and without elevated serum ALT levels are shown in Table 1. The overall prevalence of elevated ALT levels (≥40U/L) was 7.4 % (*n* = 860). Subjects with elevated serum ALT levels were more likely to be men (men, 10.5 % vs women, 4.8 %, p < 0.001), and had a higher percentage of current drinking compared with those with normal serum ALT levels. Additionally, subjects with elevated ALT levels had higher levels of SBP, DBP, and FPG, TC, TG, LDL-C, and serum uric acid levels, but lower HDL-C levels than those with normal ALT levels. Furthermore, subjects with elevated ALT levels had a heavier weight, taller height, greater waist circumference, and higher BMI compared with those with normal serum ALT levels.

**Table 1:** Characteristics of participants according to the serum levels of ALT

Variables	Total	ALT ≤ 40	ALT > 40	P value	Male	Female	P value
	(*n* = 11573)	(*n* = 10713)	(*n* = 860)		(*n* = 5357)	(*n* = 6216)	
Age (year)				<0.001			<0.001
35–44(%)	2751(23.8)	2487(23.2)	264(30.7)		1218(22.7)	1533(24.7)	
45–54(%)	3598(31.1)	3292(30.7)	306(35.6)		1621(30.3)	1977(31.8)	
55–64(%)	3481(30.0)	3257(30.4)	224(26.0)		1634(30.5)	1847(29.7)	
≥65(%)	1743(15.1)	1677(15.7)	66(7.7)		884(16.5)	859(13.8)	
Sex				<0.001			
male(%)	5357(46.3)	4794(44.7)	563(65.5)				
female(%)	6216(53.7)	5919(55.3)	297(34.5)				
Race				0.431			0.645
Han(%)	10968(94.8)	10148(94.7)	820(95.3)		5071(94.7)	5897(94.9)	
Others(%)	605(5.2)	565(5.3)	40(4.7)		286(5.3)	319(5.1)	
Current smoking status (%)	4086(35.3)	3757(35.1)	329(38.3)	0.060	3056(57.0)	1030(16.6)	<0.001
Current drinking status (%)	2617(22.6)	2333(21.8)	284(33.0)	<0.001	2434(45.4)	183(2.9)	<0.001
Education (%)				<0.001			<0.001
Primary school or below	5763(49.8)	5391(50.3)	372(43.3)		2232(41.7)	3531(56.8)	
Middle school	4717(40.8)	4325(40.4)	392(45.6)		2512(46.9)	2205(35.5)	
High school or above	1093(9.4)	997(9.3)	96(11.2)		613(11.4)	480(7.7)	
Physical activity (%)				0.886			<0.001
Low	3441(29.7)	3192(29.8)	249(29.0)		1209(22.6)	2232(35.9)	
Moderate	7479(64.6)	6912(64.5)	567(65.9)		3851(71.9)	3628(58.4)	
High	653(5.7)	609(5.7)	44(5.1)		297(5.5)	356(5.7)	
Family income (CNY/year, %)				<0.001			<0.001
≤5000	1441(12.5)	1367(12.8)	74(8.6)		720(13.4)	721(11.6)	
5000–20000	6306(54.5)	5864(54.7)	442(51.4)		2870(53.6)	3436(55.3)	
>20000	3826(33.0)	3482(32.5)	344(40.0)		1767(33.0)	2059(33.1)	
Diabetes	1199(10.4)	1074(10.0)	125(14.5)	<0.001	529(9.9)	670(10.8)	0.119
Hypertension	5914(51.1)	5409(50.5)	505(58.7)	<0.001	2889(53.9)	3025(48.7)	<0.001
Weight(kg)	64.1 ± 11.4	63.5 ± 11.0	71.5 ± 13.3	<0.001	68.6 ± 11.1	60.3 ± 10.1	<0.001
Height(cm)	160.6 ± 8.2	160.4 ± 8.2	163.5 ± 8.3	<0.001	166.4 ± 6.3	155.6 ± 6.1	<0.001
BMI(Kg/m2)	24.8 ± 3.7	24.7 ± 3.6	26.7 ± 4.2	<0.001	24.7 ± 3.5	24.9 ± 3.8	<0.001
WC(cm)	82.4 ± 9.8	82.0 ± 9.7	88.0 ± 10.1	<0.001	83.8 ± 9.8	81.3 ± 9.7	<0.001
SBP (mmHg)	141.8 ± 23.5	141.5 ± 23.5	144.8 ± 22.1	<0.001	143.7 ± 22.6	140.1 ± 24.0	<0.001
DBP(mmHg)	82.1 ± 11.8	81.7 ± 11.7	85.9 ± 12.0	<0.001	83.8 ± 11.8	80.6 ± 11.5	<0.001
FPG (mmol/L)	5.9 ± 1.6	5.9 ± 1.6	6.1 ± 1.6	<0.001	6.0 ± 1.7	5.9 ± 1.6	0.004
TC (mmol/L)	5.2 ± 1.1	5.2 ± 1.1	5.5 ± 1.3	<0.001	5.2 ± 1.0	5.3 ± 1.1	<0.001
TG (mmol/L)	1.6 ± 1.5	1.6 ± 1.4	2.3 ± 2.1	<0.001	1.7 ± 1.6	1.6 ± 1.3	0.145
LDL-C (mmol/L)	2.9 ± 0.8	2.9 ± 0.8	3.1 ± 0.9	<0.001	2.9 ± 0.8	3.0 ± 0.8	<0.001
HDL-C (mmol/L)	1.4 ± 0.4	1.4 ± 0.4	1.3 ± 0.4	<0.001	1.4 ± 0.4	1.4 ± 0.3	0.645
Serum uric acid (mg/dL)	291.9 ± 84.9	288.1 ± 82.6	338.9 ± 97.8	<0.001	333.7 ± 83.5	255.8 ± 67.8	<0.001

Data are expressed as the mean ± SD or as n (%)

Abbreviations: *CNY* China Yuan (1CNY = 0.161 USD); *SBP* systolic blood pressure; *DBP* diastolic blood pressure; *BMI* body mass index; *WC* waist circumference; *TC* total cholesterol; *TG* triglyceride; *LDL-C* low-density lipoprotein cholesterol; *HDL-C* high-density lipoprotein cholesterol; *FPG* fasting plasma glucose

### Adjusted Odd ratios of associated cardiometabolic risk factors for elevated serum ALT levels

Table 2 shows the adjusted ORs for the associations between elevated serum ALT levels and certain cardiometabolic risk factors. Subjects with elevated serum ALT levels had higher levels of almost all cardiometabolic risk factors than those with normal serum ALT levels. Participants who had elevated serum ALT levels showed a more pronounced prevalence of hypertension (adjusted OR, 1.30; 95 % CI, 1.11–1.53), hypercholesterolemia (adjusted OR, 1.57; 95 % CI, 1.26–1.96), hypertriglyceridemia (adjusted OR, 1.59; 95 % CI, 1.28–1.96), low HDL-C levels (adjusted OR, 1.27; 95 % CI, 1.03–1.55), BMI ≥25 kg/m^2^ (adjusted OR, 1.51; 95 % CI, 1.18–1.94), abdominal obesity (adjusted OR, 1.53; 95 % CI, 1.19–1.96), hyperuricemia (adjusted OR, 1.78; 95 % CI, 1.47–2.16), and HtgW phenotype (adjusted OR, 1.37; 95 % CI, 1.07–1.76) compared with those with normal serum ALT levels, after adjusting for sex, age, race, BMI, current smoking status, current drinking status, hypertension, diabetes, education, physical activity, and income.

**Table 2:** Adjusted ORs of Associated cardiometabolic risk Factors for Elevated Serum ALT Level (Q40 U/L)

Variables	OR	95 % CI	P
Age, yrs	0.96	0.95–0.97	<0.001
Sex			
female	1(referent)	一	一
male	2.98	2.44–3.64	<0.001
Race			
Han	1(referent)	一	一
Others	0.78	0.55–1.10	0.158
Current smoking status	0.73	0.62–0.86	<0.001
Current drinking status	1.12	0.93–1.35	0.233
Hypertension	1.30	1.11–1.53	0.001
diabetes	1.22	0.98–1.52	0.073
High TC	1.57	1.26–1.96	<0.001
High TG	1.59	1.28–1.96	<0.001
Low HDL	1.27	1.03–1.55	0.022
High LDL	0.89	0.60–1.34	0.592
BMI ≥ 25 kg/m2	1.51	1.18–1.94	0.001
Abdominal obesity	1.53	1.19–1.96	0.001
Hyperuricemia	1.78	1.47–2.16	<0.001
HtgW phenotype	1.37	1.07–1.76	0.012

Adjusted for age, sex, race, BMI, Current smoking status, Current drinking status, Education, Physical activity, Family income, Hypertension, diabetes, high TC, high TG, low HDL, high LDL, BMI ≥25 kg/m^2^, abdominal obesity, Hyperuricemia and HtgW phenotype

### Association between elevated ALT levels and cardiometabolic risk factors according to sex

Subsequent to adjustment for confounding factors, the data showed dramatically different results in multiple logistic regression according to sex (Table 3). For men, cardiometabolic risk factors that were associated with elevated ALT levels included age (OR,0.95; 95 % CI, 0.94–0.96), current smoking status (OR,0.62; 95 % CI, 0.52–0.75), hypertension (adjusted OR, 1.33; 95 % CI, 1.08–1.62), hypercholesterolemia (adjusted OR, 1.63; 95 % CI, 1.23–2.17), hypertriglyceridemia (adjusted OR, 1.62; 95 % CI, 1.25–2.09), BMI ≥25 kg/m^2^ (adjusted OR, 1.52; 95 % CI, 1.07–2.18), and hyperuricemia (adjusted OR, 1.92; 95 % CI, 1.54–2.40). However, for women, cardiometabolic risk factors that were significantly related to elevated ALT levels included age (OR, 0.98; 95 % CI, 0.97–0.99), hypertriglyceridemia (adjusted OR, 1.74; 95 % CI, 1.30–2.33), BMI ≥25 kg/m^2^ (adjusted OR, 1.81; 95 % CI, 1.27–2.58), and abdominal obesity (adjusted OR, 1.75; 95 % CI, 1.30–2.35).

**Table 3:** Association between elevated ALT level and cardiometabolic risk factors according to the sex

Variables	Male			Female		
	OR	95 % CI	P	OR	95 % CI	P
Age, yrs	0.95	0.94–0.96	<0.001	0.98	0.97–0.99	0.009
Race						
Han	1(referent)		0.321	1(referent)		0.415
Others	0.78	0.51–1.19	0.274	0.73	0.40–1.33	0.309
Current smoking status	0.62	0.52–0.75	<0.001	1.06	0.76–1.48	0.733
Current drinking status	1.12	0.92–1.32	0.267	1.07	0.53–2.18	0.842
Hypertension	1.33	1.08–1.62	0.006	1.22	0.93–1.61	0.148
diabetes	1.21	0.91–1.61	0.192	1.23	089–1.76	0.195
High TC	1.63	1.23–2.17	0.001	1.38	0.96–1.99	0.084
High TG	1.62	1.25–2.09	<0.001	1.74	1.30–2.33	<0.001
Low HDL	1.23	0.96–1.58	0.096	1.33	0.93–1.89	0.116
High LDL	0.91	0.61–1.36	0.640	1.21	0.78–1.89	0.393
BMI ≥ 25 kg/m2	1.52	1.07–2.18	0.021	1.81	1.27–2.58	0.001
Abdominal obesity	1.22	0.78–1.90	0.377	1.75	1.30–2.35	<0.001
Hyperuricemia	1.92	1.54–2.40	<0.001	1.34	0.90–1.99	0.145
HtgW phenotype	1.32	0.97–1.79	0.077	1.500	0.97–2.00	0.067

Adjusted for age, race, BMI, Current smoking status, Current drinking status,Education,Physical activity, Family income, Hypertension, diabetes, high TC, high TG, low HDL, high LDL, BMI ≥25 kg/m^2^, abdominal obesity, Hyperuricemia and HtgW phenotype

## Discussion

To the best of our knowledge, the present study is the first large-scale, population-based, cross-sectional survey to report the relationships between elevated serum ALT levels and cardiometabolic risk factors in a rural area of China.

Elevated serum ALT levels are most closely related to liver fat accumulation and are commonly used as a surrogate marker for nonalcoholic fatty liver disease in epidemiological studies [22, 24]. Our study examined significant associations of elevated serum ALT levels with cardiometabolic risk factors in a rural Chinese population. After adjustment for various confounders, most of these associations remained significant. Sex and age-adjusted current smoking were significantly related to a lower likelihood of elevated serum ALT levels. Although current smoking might constitute an important risk factor with regard to liver function abnormalities [25], this is not surprising because smoking lowers the risk of type-2 diabetes, presumably by inhibiting autoimmune processes [26]. Previous studies have found that elevated ALT levels are associated with obesity and metabolic syndrome, including a study performed in the Japanese population [27] and a population-based cross-sectional survey in Korean adolescents [28].

The central characteristics of metabolic syndrome are hypertension, hyperglycemia, dyslipidemia, and abdominal obesity. Consistent with the results of other epidemiological studies [27, 29], our study showed positive associations between elevated serum ALT levels and cardiometabolic risk factors, such as hypertension, hypercholesterolemia, hypertriglyceridemia, low HDL-C levels, and abdominal obesity. Diabetes is inversely predicted by lipoprotein(a) [30–32] and is strongly predicted by HtgW, which is also inversely predicted by lipoprotein(a). ALT is assumed to be collinear with Lp(a) and thereby acts as a mediator for cardiovascular risk factors, including the HtgW phenotype. However, an association between elevated ALT levels and diabetes mellitus was not found in our study.

In addition, we investigated the effect of sex on the associations of elevated serum ALT levels with cardiometabolic risk factors in a rural Chinese population. The overall prevalence of elevated ALT levels in our study (7.4 % for men and women; 10.5 % and 4.8 %, respectively) is similar to a previous population-based study that was conducted in the general Chinese population [2], but is much lower than another related study in China [33]. Multiple logistic regression analysis indicated that male sex, a younger age, the presence of hypertension, hypercholesterolemia, hypertriglyceridemia, low HDL-C levels, BMI ≥25 kg/m^2^, abdominal obesity, hyperuricemia, and the HtgW phenotype were independent predictors of an elevated ALT level. Unsurprisingly, male sex and a younger age represented significant risk factors that were related to the likelihood of an elevated serum ALT level. These results are consistent with the results of other studies [9, 34]. Elevated serum ALT levels among predominantly younger male adults remain unexplained and deserve further attention [9]. Although our results relating to the significant association between elevated serum ALT levels and hyperuricemia appear to be similar to those of previous studies, the extent of elevation seems to be dependent on the severity of the hepatic lesions [2, 35]. Based on the sex-related data, differences in both sexes were present in the relationships between elevated serum ALT levels and cardiometabolic risk factors. For men, we found that elevated ALT levels were closely associated with age, current smoking status, hypertension, hypercholesterolemia, hypertriglyceridemia, BMI ≥25 kg/m^2^, and hyperuricemia. However, for women, elevated ALT levels were mainly associated with age, hypertriglyceridemia, BMI ≥25 kg/m^2^, and abdominal obesity.

Although current drinking status might be an important risk factor with regard to abnormalities of liver function in other population-based studies [2, 34], we did not find any significance for this risk factor in our study. In this sample of the rural Chinese population, we did not observe an association between elevated serum ALT levels and physical activity, even after adjustment for age and sex. To the best of our knowledge, data on the association between physical activity and serum ALT levels are limited. An inverse association between physical activity and serum ALT levels among middle-aged British women [11] and obese children [36] has been reported.

Several limitations in this study need to be acknowledged. First, because of the cross-sectional design of our study, we were unable to determine whether there was a causal association between elevated ALT levels and cardiometabolic risk factors. Therefore, the obtained associations in this study should be considered with caution. Second, despite extensive adjustment in our study, unmeasured confounders may explain part of the association between elevated ALT levels and cardiometabolic risk factors. There are known causes of elevated ALT levels that were not tested in our study, such as alcohol abuse, chronic viral hepatitis, and other illnesses. However, the strengths of this study are its population-based design, large sample size, and extensive information on confounders.

## Conclusions

In conclusion, there is a significant association between elevated ALT levels and cardiometabolic risk factors in rural Northeast China in both sexes. Individuals with elevated ALT levels have a higher risk of cardiometabolic abnormalities than those with normal ALT levels. Therefore, elevated serum ALT levels are associated with a worse cardiac risk profile. Further prospective studies are required to verify these findings.
